# Cancer

**Published:** 1906-01-27

**Authors:** 


### CANCER.

**The Growth of Cancer.**—Two aspects of the problem of cancer may be summed up in the questions "What is cancer?" and "What produces cancer?" The latter question has been the subject of numerous investigations and theories, but possibly the former is the one to which scientists should first address themselves; at any rate, this is the opinion of the General Superintendent of the work of the Cancer Research Fund. The investigations undertaken by Bashford [1] have for their object the determination of the characteristics of malignant growth, and in this way he hopes that light may be thrown on the cause or causes which go to produce these characteristics. So far the results of two years' work have led him to the following conclusions: From the negative side cancer does not resemble any known form of infective or contagious disease; the only way in which cancer can be transmitted from one individual animal to another is by transference of living cancer cells in a way which permits of their continued growth. If under natural conditions cancer is always so produced, an enormous number of different varieties of cancer cells must be supposed to have originated in the distant past and to be transmitted, each to its appropriate host and organ, by direct infection from a previous case. This theory Bashford considers untenable, especially in view of the age incidence of cancer. From the positive side the reseach so far has shown that cancer cells from an animal are only directly transmissible to another of the same species, and that in all animals cancer is a condition of later life—that is, that its age incidence in all animals bears a similar ratio to the average duration of life. With regard to particular organs the same holds true, as those organs which become functionally old earlier in life, such as the generative organs, have an earlier age incidence as regards cancer, whereas those which, like the skin, retain their functional activity longer are attacked later by the disease. The next point Bashford considers proved is that cancer cells are formed by the growth of previous cancer cells, and that the tumours so formed are not composed of the altered tissue elements of the animal, as far as their parenchyma is concerned. The connective tissue stroma is, however, formed from the tissues of the part invaded, modified in some way by the presence of the parenchymatous cancer cells. As the result of a large number of transplantations in mice, Bashford concludes that the only constant characteristic of cancer cells is their power of unlimited growth: the other symptoms, such as cachexia, did not appear under experimental conditions. The investigation of the minute processes of growth—that is, the microscopic changes in the cells and their nuclei—gave rise to some interesting conclusions. The cells divide in various ways, both directly and indirectly, the latter method being the more prominent. Not infrequently abnormal mitosis occurs, such as division of the nucleus into several spindles converging towards a corresponding number of poles. Both the ordinary bipolar division and this multipolar division are also sometimes asymetrical. All these variations occur also in benign tumours and in chronic inflammatory tissue. There is, however, one form of mitosis which is peculiar to malignant growths, though not constantly occurring. It is that termed heterotype mitosis, and is similar to, though not identical with, that normally occurring in reproductive tissues. Now, although in cases of cancer these various forms of cell division appear confused and unintelligible, it was found that in experimentally-produced growths cell division invariably occurred in a regular and ordinary way, but that at certain regular intervals, and in connection with other phenomena, "heterotype" mitosis occurred. Heterotype mitosis in animals and plants marks a terminal phase in cell proliferation, preparatory to the sexual production, by fusion of nuclei, of a new generation. Provisionally, as a working hypothesis, Bashford concludes that occasionally the cells of organs tend to undergo changes (heterotype mitosis) which prepare them for nuclear fusion. This nuclear fusion, however, is only rarely attained, as, unlike normal reproductive tissues, there is no special mechanism to ensure its occurrence. When it *does* occur, then a new cycle of growth begins in the cells of the organ, constituting them, as it were, a new generation with individual powers of growth along a particular line—namely, that of the original cells from which they arise. This at present explains the age incidence (senility) and all other features of malignant growth, but it requires confirmation in the light of future experiment and experience.

**Confirmatory views** of this theory seem to be held by certain other observers. Orth,[2] for instance, in regarding the cell as the infective agent as far as our present knowledge goes, and Hausemann,[3] in his remarks on the transplantation of malignant tumours, uphold the view supported by Bashford that the parenchyma cells of cancers are derived by division of previous cells, and not by mutation of the cells of the host. All they derive from the latter is nourishment afforded by stroma cells and vessels, which, however, as Orth points out, are not invariably present. Cooper,[4] in a note commenting on Bashford's hypothesis, points out that it is on similar lines to one he enunciated in 1900, before the phenomena of heterotype mitosis had been described. He considers, however, that, as it stands, the hypothesis does not explain the fact that the cells of the very malignant sarcomata are undifferentiated. He would account for this by supposing a failure of function in newly-formed cells, whereby they cease to be able to differentiate; hence, their capacity for abnormally active growth may be imagined to be increased. With regard to already differentiated cells, as in epithelioma, he points out that when some sort of nuclear fusion (comparable to fertilisation) has taken place, the daughter cells must be supposed to show diminished function with increased gain in reproductive activity, whereas in normal reproductive tissue differentiation and increase of function go on *pari passu* with proliferation. Wilson[5] draws attention to the fact that, in addition to the characteristic of limitless growth, cancerous tumours are devoid of nerves. The view that cancer cells are functionless and independent of the body needs is, he thinks, supported by this fact and by certain chemical differences established by Blumenthal. He points out the analogy between masses of cancer cells and certain cell communities or colonies of infusoria and rhizopods, and says that most forms of cell-division found in cancers have also been observed in protozoa. Loss of function or involution of the cell, whether induced by senility or some morbid process, is the preparation for cancerous growth, as the cell then tends to return to the more primitive function of nerve growth. He also suggests that in higher animals nerve influence by withdrawing energy for specialised function may inhibit the primitive tendency to unlimited cell reproduction. When, owing to the highly specialised nature of nerve influence it becomes very liable to derangement of function, it may lose some control over the growth of epithelial cells, which, as a result of local irritation, may then proliferate in a primitive matter causing a cancerous tumour. He thinks the fact brought out by Bashford that cancers can only be successfully transplanted into animals of the same species implies a nutritional factor in their growth. It may also, as Bashford sugests, imply the existence of antibodies analogous to hæmolysins.

[1] Brit. Med. Jour., July 8, 1905, p. 96. Lancet. Ap.1, 1905, p 836. [2] Med. Rec., May 6, 1905, p. 699. [3] Ibid. [4] Med. Chron., April, 1905, p. 99. [5] Lancet, June 10, 1905, p. 1611.

*(To be continued.)*

